# Commentary: Decoding the Charitable Brain: Empathy, Perspective Taking, and Attention Shifts Differentially Predict Altruistic Giving

**DOI:** 10.3389/fnbeh.2017.00110

**Published:** 2017-06-07

**Authors:** Vanessa Era, Martina Fusaro, Selene Gallo

**Affiliations:** ^1^Social Cognitive Neuroscience Laboratory, Department of Psychology, Sapienza University of RomeRome, Italy; ^2^Social Cognitive Neuroscience Laboratory, Fondazione Santa Lucia, IRCCSRome, Italy; ^3^Social Brain Lab, The Netherlands Institute for Neuroscience, Royal Netherlands Academy of Arts and Sciences (KNAW)Amsterdam, Netherlands

**Keywords:** empathy, perspective taking, STORM model, mPFC, TPJ, prosocial behavior

Altruism is acting to promote someone else's welfare, even at a risk or cost to ourselves. In altruistic decision making the emotional sharing of the other person's affective state needs to be integrated with cognitive evaluations of the social context in which the decision takes place. More specifically, in the case of charitable donation the donor needs to evaluate the mission of the charity in order to make an informed decision. In a recent paper by Tusche et al. (2016), the authors explored the specific contribution of empathy and perspective taking (PT) in altruistic behavior. Empathy is the capacity to understand the affective experience of another person (Decety and Jackson, 2004) while PT is the cognitive process of inferring others' thoughts and intentions (Frith and Frith, 2006). The paper focused on disentangling the specific role of empathy and PT and of their neural correlates, anterior insula (AI), and the temporo-parietal junction (TPJ), respectively, in altruistic behavior.

In this commentary, we discuss Tusches' and colleagues interesting results, paying special attention to the role of prefrontal areas in higher-level cognitive processes involved in the charitable decision-making process. In their study, the authors asked participants to donate money to different charities during fMRI. Participants were later asked to rate how processes previously associated with altruistic behavior, including empathic responses and PT, characterized each charity. An additional task was performed to independently measure participants' empathy and PT neural correlates (EmpaToM, Kanske et al., 2015). Activity in different areas, including those traditionally associated with affective sharing (AI), PT (TPJ), and decision making (ventromedial and dorsolateral prefrontal cortices), distinguished between selfish and generous donation.

The authors focused on the finding of a segregated role of TPJ and AI in supporting donations promoted by PT and empathy, respectively. Interestingly, medial prefrontal cortex (mPFC) activity was found across tasks. Firstly, the activity of this region distinguished between generous and selfish donation. Secondly, its activity coded for degrees of empathy (but not PT) toward the charities when the authors disentangled the contribution of empathy and PT to donation trial-by-trial. Finally, mPFC activity predicted donation generosity supported by PT (but not by empathy) when looking at its activity during EmpaToM. Not being the focus of their study, the authors did not clarify these ambiguous results about the involvement of the prefrontal cortices in altruistic giving. Charitabile donations are shaped by moral motivations, evaluations of risks and costs, guilt, personal values, prospective evaluations, and the ability to understand another person (Batson, 1989). This latter ability might be supported by affectively resonating with the other (empathy) or by cognitively evaluating other's state (PT). Both of these processes might be modulated by the beneficiary of the donation (social context). Thus, investigating the role of the prefrontal cortices in charitable donations can also clarify the relationship between empathy and PT in this process.

Tusche et al. showed that the activation of mPFC supported both empathy's and PT's contribution to donation. However, this activation was not consistent across different tasks. The technique used in the study might have underestimated the shared modulation of empathy and PT by mPFC in supporting donations. More specifically, the technique employed used a linear decision boundary applied to a small local cluster of voxels. Multi Voxel Pattern Analysis (MVPA) is in general more sensitive to high frequency spatial difference between conditions than a univariate general linear model (GLM) approach. This feature was fundamental in finding distinct neural underpinnings for empathy and PT in different regions of the brain supporting donations, a task in which the GLM failed. It is important to note that classification algorithms tend to focus on discriminative features and ignore features that are shared across the categories. Tusche et al. tested whether local clusters in specific regions represented empathy and/or PT. They found that areas having information regarding one of the two processes did not contain information about the other in supporting altruism. This does not exclude that the relationship between covariance of those areas, or between those areas and control ones, carries the information about the degree in which empathy and PT influence charitable donation.

Social contexts might, in fact, influence the recruitment of empathy and PT, contributing to the flexibility of the altruistic behavior. This context-based top-down modulation of social behaviors (Lakin et al., 2003) has been proposed to rely on a specific brain network in which mPFC is an important node (Wang et al., 2011). Wang and Hamilton (2012) proposed a model regarding the control of imitative behavior: the social top-down response modulation (STORM). The STORM model assumes that mimicry plays an important role in communication and affiliation and makes individuals more prone to be engaged in positive social interactions (Lakin and Chartrand, 2003; Lakin et al., 2003). Spontaneous mimicry is modulated by social signals requiring the engagement of empathy and perspective taking processes (Chartrand et al., 2005). The same processes also seem to be involved in prosocial behavior, such as charitable donations (Mathur et al., 2010; Telzer et al., 2011; Morelli et al., 2014). For this reason we suggest that the STORM model could also explain the modulation of charitable donation based on social context. Imitation is guided by social cues (Lakin and Chartrand, 2003), and the same cues could also guide prosocial behavior (i.e., group diversity, Andreoni et al., 2016). More specifically, the STORM model assumes that imitative behavior is modulated by social context. Responses evoked in the mirror neuron system are subjected to top-down control. This control mechanism is supported by the activity of mPFC. The modulation is based on an evaluation of both the current context and the social situation. We suggest that a similar top-down mechanism, in which empathy and PT are not two independent processes but are controlled together by mPFC, might be generalized to the modulation of altruistic donation, where the social context is represented by the beneficiary of the prosocial behavior.

The involvement of prefrontal regions in altruistic behavior has been highlighted in many studies (Hare et al., 2010; Waytz et al., 2012). Tusche et al. found that the recruitment of empathy and PT predicting altruistic behavior is flexible and dependent on the different charitable organizations (social context) to which participants were asked to donate. This contextual influence can be interpreted in light of the STORM model. Prefrontal cortices might evaluate different charities (social context) and modulate the amount of empathy or PT recruited, resulting in different levels of generosity. We highlighted the role of prefrontal areas in higher-level cognitive processes involved in the charitable decision-making process. Although empathy and PT support charitable donation separately as showed by Tusche et al., it is also important to consider that both of them are modulated by the same high-level regions. We suggest that those regions preferentially support PT or empathy depending on the social context, and also modulate the degree to which PT and empathy are engaged to promote altruistic choices that represent a social reward.

## Author contributions

VE, MF, and SG have made substantial, direct, and intellectual contribution to the work and approved it for publication.

### Conflict of interest statement

The authors declare that the research was conducted in the absence of any commercial or financial relationships that could be construed as a potential conflict of interest.

## References

[B1] Andreoni J. Payne A. A. Smith J. Karp D. (2016). Diversity and donations: the effect of religious and ethnic diversity on charitable giving. J. Econ. Behav. Organ. 128, 47–58. 10.1016/j.jebo.2016.05.010

[B2] Batson C. D. (1989). Personal values, moral principles, and a three-path model of prosocial motivation, in Social and Moral Values: Individual and Societal Perspectives, eds Eisenberg N. Reykowski J. Staub E. (Mahwah, NJ: L. Erlbaum Associates), 213–228.

[B3] Chartrand T. L. Maddux W. W. Lakin J. L. (2005). Beyond the perception-behavior link: The ubiquitous utility and motivational moderators of nonconscious mimicry, in The New Unconscious, eds Hassin R. R. Uleman J. S. Bargh J. A. (New York, NY: Oxford University Press), 334–361.

[B4] Decety J. Jackson P. L. (2004). The functional architecture of human empathy. Behav. Cogn. Neurosci. Rev. 3, 71–100. 10.1177/1534582304267187 15537986

[B5] Frith C. D. Frith U. (2006). The neural basis of mentalizing. Neuron 50, 531–534. 10.1016/j.neuron.2006.05.001 16701204

[B6] Hare T. A. Camerer C. F. Knoepfle D. T. O'Doherty J. P. Rangel A. (2010). Value computations in ventral medial prefrontal cortex during charitable decision making incorporate input from regions involved in social cognition. J. Neurosci. 30, 583–590. 10.1523/JNEUROSCI.4089-09.2010 20071521 PMC6633003

[B7] Kanske P. Böckler A. Singer T. (2015). Models, mechanisms and moderators dissociating empathy and Theory of Mind, in Social Behavior from Rodents to Humans: Neural Foundations and Clinical Implications, Current Topics in Behavioral Neurosciences, eds Wöhr M. Krach S. (Luxemburg: Springer), 1–14. 10.1007/7854_2015_412 26602249

[B8] Lakin J. L. Chartrand T. L. (2003). Using nonconscious behavioral mimicry to create affiliation and rapport. Psychol. Sci. 14, 334–339. 10.1111/1467-9280.14481 12807406

[B9] Lakin J. L. Jefferis V. E. Cheng C. M. Chartrand T. L. (2003). The chameleon effect as social glue: evidence for the evolutionary significance of nonconscious mimicry. J. Nonverbal Behav. 27, 145–162. 10.1023/A:1025389814290

[B10] Mathur V. A. Harada T. Lipke T. Chiao J. Y. (2010). Neural basis of extraordinary empathy and altruistic motivation. Neuroimage 51, 1468–1475. 10.1016/j.neuroimage.2010.03.025 20302945

[B11] Morelli S. A. Rameson L. T. Lieberman M. D. (2014). The neural components of empathy: predicting daily prosocial behavior. Soc. Cogn. Affect. Neurosci. 9, 39–47. 10.1093/scan/nss088 22887480 PMC3871722

[B12] Telzer E. H. Masten C. L. Berkman E. T. Lieberman M. D. Fuligni A. J. (2011). Neural regions associated with self control and mentalizing are recruited during prosocial behaviors towards the family. Neuroimage 58, 242–249. 10.1016/j.neuroimage.2011.06.013 21703352 PMC3276247

[B13] Tusche A. Böckler A. Kanske P. Trautwein F. M. Singer T. (2016). Decoding the charitable brain: empathy, perspective taking, and attention shifts differentially predict altruistic giving. J. Neurosci. 36, 4719–4732. 10.1523/JNEUROSCI.3392-15.2016 27122031 PMC6601722

[B14] Wang Y. Hamilton A. F. (2012). Social top-down response modulation (STORM): a model of the control of mimicry in social interaction. Front. Hum. Neurosci. 6:153. 10.3389/fnhum.2012.00153 22675295 PMC3366585

[B15] Wang Y. Ramsey R. Hamilton A. F. (2011). The control of mimicry by eye contact is mediated by medial prefrontal cortex. J. Neurosci. 31, 12001–12010. 10.1523/JNEUROSCI.0845-11.2011 21849560 PMC6623188

[B16] Waytz A. Zaki J. Mitchell J. P. (2012). Response of dorsomedial prefrontal cortex predicts altruistic behavior. J. Neurosci. 32, 7646–7650. 10.1523/JNEUROSCI.6193-11.2012 22649243 PMC3387686

